# Online 3D Ear Recognition by Combining Global and Local Features

**DOI:** 10.1371/journal.pone.0166204

**Published:** 2016-12-09

**Authors:** Yahui Liu, Bob Zhang, Guangming Lu, David Zhang

**Affiliations:** 1 Department of Computer Science and Technology, Harbin Institute of Technology Shenzhen Graduate School, Shenzhen, China; 2 Department of Computer and Information Science, University of Macau, Macau; National University of Defense Technology College of Mechatronic Engineering and Automation, CHINA

## Abstract

The three-dimensional shape of the ear has been proven to be a stable candidate for biometric authentication because of its desirable properties such as universality, uniqueness, and permanence. In this paper, a special laser scanner designed for online three-dimensional ear acquisition was described. Based on the dataset collected by our scanner, two novel feature classes were defined from a three-dimensional ear image: the global feature class (empty centers and angles) and local feature class (points, lines, and areas). These features are extracted and combined in an optimal way for three-dimensional ear recognition. Using a large dataset consisting of 2,000 samples, the experimental results illustrate the effectiveness of fusing global and local features, obtaining an equal error rate of 2.2%.

## 1. Introduction

Biometric authentication is of great importance for applications in public security [1, 2, 3]. Nowadays, several novel biometrics, including palmprints [4], veins [5], and ears [6–12], have been developed to meet the needs of different security requirements.

With advances in three-dimensional (3D) imaging technology, 3D biometric authentication has drawn increasing attention from researchers. Examples include 3D face [13, 14], palmprint [15–17], and ear recognition [18–23]. A 3D ear image is robust to imaging conditions, and contains surface shape information that is related to anatomical structure. In addition, it is insensitive to environmental illuminations. Yan and Bowyer [18] utilized both color and depth images to determine the ear pit for automated 3D ear segmentation. Furthermore, they proposed an improved Iterative Closest Point (ICP) algorithm for 3D ear point cloud matching. Chen and Bhanu [19] gave a 3D ear recognition method founded on a Local Surface Patch (LSP) and ICP algorithm. Moreover, they proposed an indexing approach [20] that combines feature embedding and a support vector machine-based learning technique for ranking their hypotheses. Islam et al. presented a local 3D features extraction method based on the key point detection [21, 22]. Zhou et al. presented a 3D ear recognition system combining local and holistic features [23]. Zhang et al. introduced a sparse representation framework into the field of 3D ear identification [24]. Chen and Mu proposed a hybrid multi-keypoint descriptor sparse representation-based classification (MKD-SRC) method to solve one sample per person problem in ear recognition [25].

Even though good results were achieved in these studies, there is no overall system for online 3D ear recognition. First, most of the current methods use commercial laser scanners to acquire the 3D range image, for example, the widely used Minolta VIVID Series [18–25]. Although these scanners are general-purpose and high-performance, they are expensive and cumbersome. Second, previous 3D ear recognition methods focused on a single aspect, that is, mostly local features, while global features such as the ear-parotic area angle, and the ear hole shape have not been discussed or used. Given these considerations, a laser scanner specifically designed for 3D ear acquisition and recognition was first developed using the laser-triangulation principle. The scanner provides 2D intensity images and 3D point-cloud data for subsequent recognition, and the total scanning and transmission time is less than 2 s. Based on the 3D ear images collected by our laser scanning device, two feature classes consisting of five features were defined. The empty center shape and the angle feature represent the depth and orientation of a 3D ear, and are treated as global features. The point, line, and area features describe key points, shapes, and the local area of the 3D ears. They are treated as local features. By combining these global features with local features, a hierarchical structure was introduced for 3D ear recognition. The 3D ears are pre-classified using global features and then recognized using local features. Thus, much time can be saved and accuracy can be improved in 3D ear recognition. Therefore, the 3D ear recognition system achieves both a high efficiency and accuracy.

The purpose of this study was to create a 3D ear recognition system using equipment that is practical for real applications. The contributions of this paper can be summarized as follows. Firstly, the global and local features categories in 3D ear are proposed. Secondly, multi-forms of features in 3D ears have been defined and extracted. Thirdly, multi-features fusion and hierarchical recognition of 3D ears have been discussed. Finally, a complete solution for 3D ear authentication has been achieved. The results on the collected 3D ear data show that the system is efficient and accurate.

## 2. Special Scanner Design for Online 3D Ear Acquisition

**Fig 1:**
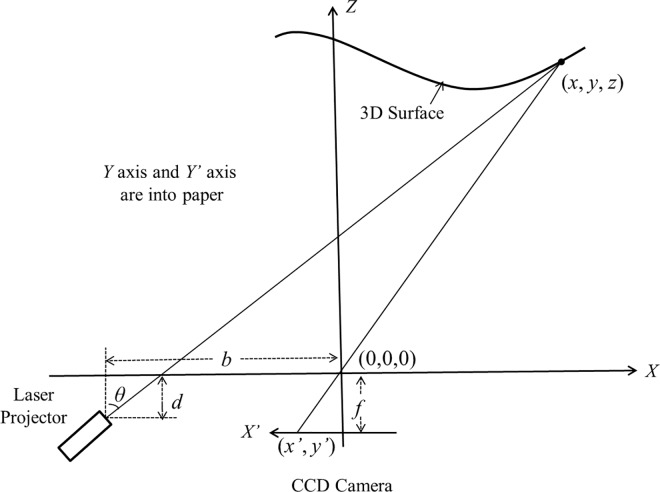
Imaging principle of laser-triangulation imaging.

The 3D ear scanner we developed is based on the laser trangulation princple [26]. Fig 1 illustrates the imaging principle of laser triangulation. In the reference *X-Y-Z* coordinates, the 3D coordinates (*x*, *y*, *z*) can be calculated according to Eq (1).

$$\vec{p}=\left(\begin{array}{c} x\\ y\\ z \end{array}\right)=\left(\begin{array}{c} \frac{x'(b-d\tan\theta )}{x'+f\tan\theta }\\ \frac{y'(b-d\tan\theta )}{x'+f\tan\theta }\\ \frac{f'(b-d\tan\theta )}{x'+f\tan\theta } \end{array}\right)$$ (1)

**Fig 2:**
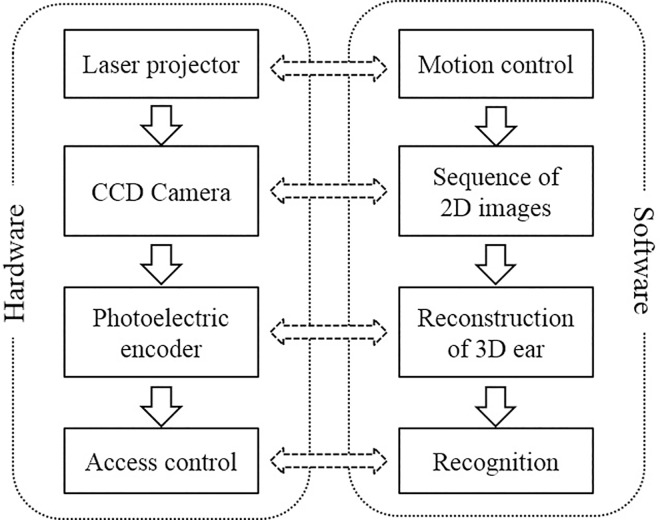
Framework of the 3D ear recognition system.

**Fig 3:**
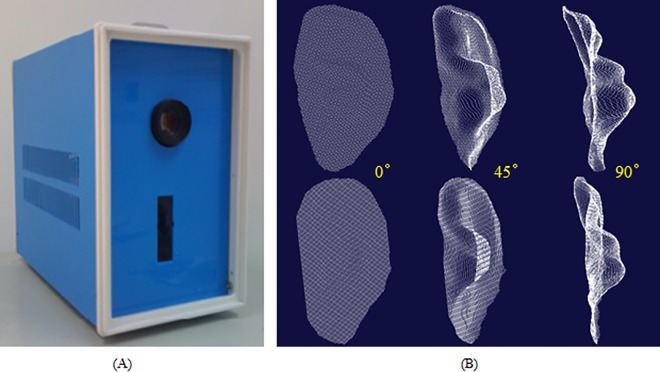
Proposed 3D ear acquisition system: (A) 3D ear acquisition device and (B) 3D ear samples viewed at different angles (each row is collected from a single ear).

Fig 2 illustrates the framework of the 3D ear recognition system. The system consists of two main parts: hardware and software. To meet the requirements of online recognition, the hardware and software should be optimized for speed and accuracy. At the same time, its portability and cost for real applications should be considered. The laser scanner developed for 3D ear acquisition is shown in Fig 3A. Fig 3B shows two group of typical 3D ear samples captured by our device, where each row is the 3D point cloud from one ear viewed at different angles.

Table 1 provide a performance comparison of our proposed device with the Minolta Vivid 910 range scanner that is a widely used commercial scanner and has been used to acquire 3D ear data for UND data set. The acquisition time refers to the total scanning and transmission time, accuracy refers to the depth precision of the measurement, dimensions refer to the width, height and length of the scanner, in addition, the weight and price are also listed. Although the measurement accuracy of our acquisition system is inferior to that of Vivid 910, it has a higher speed, smaller size, and much lower cost. Moreover, the device could provide original frames of laser lines that describe the fundamental structure of 3D features. All these traits make the specially designed device suitable for 3D ear acquisition in practical biometrics applications.

**Table 1:** Comparison of the scanning device.

	Acquisition Time (s)	Accuracy (mm)	Dimensions (mm)	Weight (kg)	Price (USD)
Vivid 910	4	±0.1	213 × 413 × 271	11	> 20,000
Our Scanner	2	±0.5	140 × 200 × 200	3	< 1,000

A 3D ear database was established using the developed 3D ear acquisition device by collecting 3D ears on two separate occasions separated by an interval of around one month. On each occasion, the subject was asked to provide two samples. The database contains 2,000 samples from 500 volunteers consisting of 341 males and 159 females. The volunteers were students and staff of the Shenzhen Graduate School of Harbin Institute of Technology. The written consents were obtained from the participants prior to the study. The study was approved by the Academic Committee of the Department of Computing of Harbin Institute of Technology, Shenzhen Graduate School, which ensures that research programs are consistent with academic ethics. The 3D ear acquisition study was discussed in a meeting of the committee, and written approval was subsequently granted by the Department Head. Because our research work does not involve patients or privacy, and all the participants have given written consent to the use of their ear images for academic purposes, all the data and figures published in this paper are fully available from the figshare.com and Biometrics Research Center of The Hong Kong Polytechnic University. Interested researchers can download the 3D ear database from following URLs: http://dx.doi.org/10.6084/m9.figshare.1378463 or http://www4.comp.polyu.edu.hk/~biometrics/. The database is used for feature extraction and recognition in the following sections.

## 3. 3D Ear Global and Local Feature Classes

Prior to feature extraction, the 3D ears were normalized using a projection density method [27]. After that, a 3D image of the ear is formed as a normalized posture in unified *X-Y-Z* coordinates, where all features are extracted from the 3D point cloud of the ear.

### 3.1 Global Feature Class

Two global features, empty center and angle, are defined in the proposed system.

#### 3.1.1 Empty Center Feature

**Fig 4:**
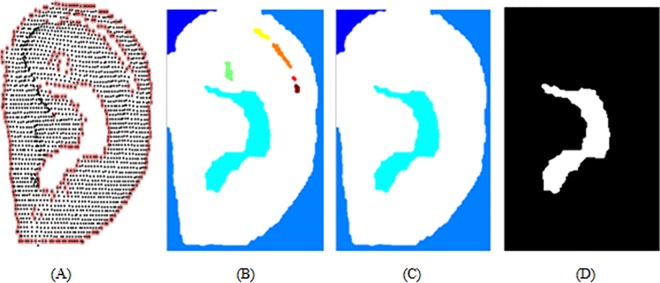
Empty center feature extraction.

In the normalized *X-Y* coordinates, the boundary points of the ear were first detected (Fig 4A), then the connected areas were labeled (Fig 4B). The connected areas that are less than a threshold were removed then (Fig 4C). Lastly, the connected pixels inside the ear were selected as the empty center feature (Fig 4D).

**Fig 5:**
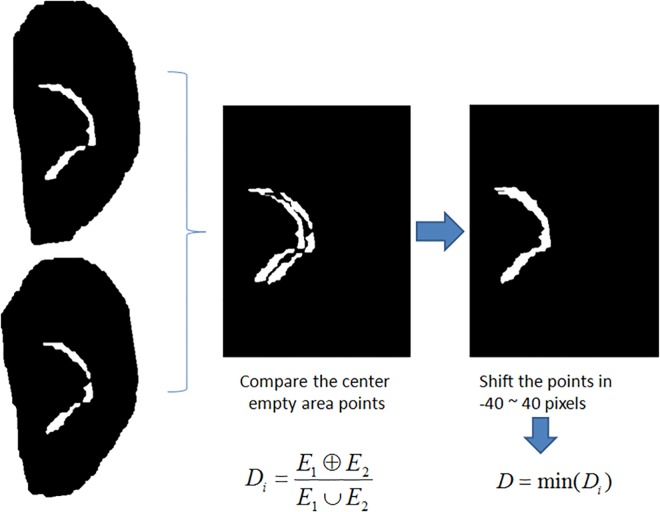
Matching empty center features.

The template matching technique is used to calculate the distance between two empty center features. The distance is defined as: *D* = *E*_1_⨁*E*_2_/*E*_1_ ∪ *E*_2_, where *E*_1_ and *E*_2_ are the empty center features of different samples. To avoid displacement interference, the test image was shifted by ±40 pixels left-right and up-down, where the minimum distance is taken to be the difference of the two empty center areas (Fig 5).

**Fig 6:**
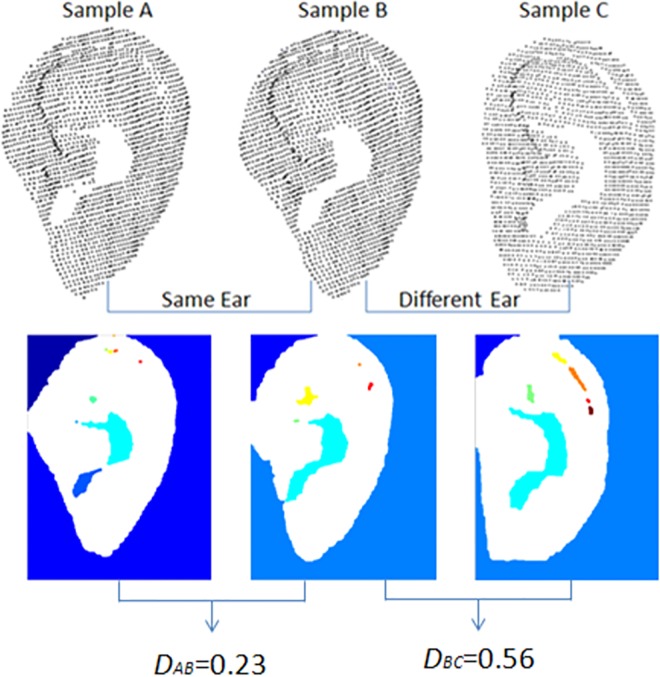
Discriminating the same ear and different ears using the empty center feature vector.

Fig 6 shows the empty center feature vectors extracted from Sample A, Sample B, and Sample C. Sample A and Sample B are from the same ear, and Sample C is from a different ear. The distance between Sample A and Sample B is 0.23, and the distance between Sample B and Sample C is 0.56, which indicates that the empty center feature vectors from the same ear are alike and those from different ears are dissimilar.

#### 3.1.2 Angle Feature

**Fig 7:**
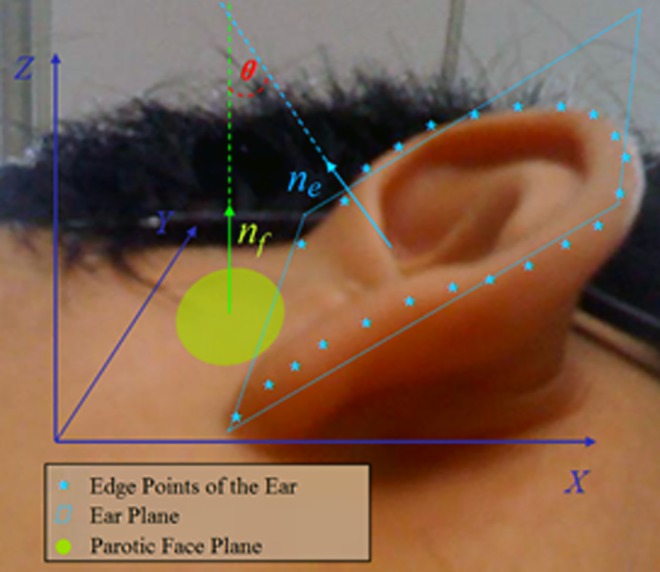
Angle feature extraction.

**Fig 8:**
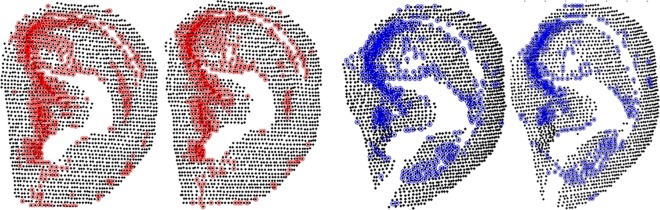
Detected key-points: The left two samples are from one ear and the other two samples are from a different ear. The detected key-points are marked by red and blue respectively.

In Fig 7, there is an angle between the ear and parotic area of a person [28]. It can be assumed that there is a plane, *A*_*f*_*x* + *B*_*f*_*y* + *C*_*f*_*z* + *D*_*f*_ = 0, which represents the 3D points on the parotic region (green circle shown in Fig 8). And there is another plane, *A*_*e*_*x* + *B*_*e*_*y* + *C*_*e*_*z* + *D*_*e*_ = 0, represents the 3D points on the ear edge. Thus, the normal vector of the parotic plane can be obtained as *n*_*f*_ = (*A*_*f*_,*B*_*f*_,*C*_*f*_), and the normal vector of the ear plane is *n*_*e*_ = (*A*_*e*_,*B*_*e*_,*C*_*e*_). The angle *θ* between the parotic and ear planes can be defined as follows:

$$\theta_{1}=arccos(\frac{<n_{f},n_{e}>}{{\left\|n_{f}\right\|}_{2}{\left\|n_{e}\right\|}_{2}})$$ (2)

Where <*n*_*f*_,*n*_*e*_> is the inner product of normal vectors *n*_*f*_ and *n*_*e*_. The ‖*n*_*f*_‖_2_ and ‖*n*_*e*_‖_2_ are L_2_-norms of *n*_*f*_ and *n*_*e*_ respectively.

Hence,

$$\theta =\left\{ \begin{array}{c} \theta_{1}\;\mathrm{if}\;\theta_{1}<90{}^{\circ}\\ 180{}^{\circ}-\theta_{1}\;\mathrm{otherwise} \end{array}\right.$$ (3)

### 3.2 Local Feature Class

Three categories of local features in the 3D ear image were defined: point, line, and area features.

#### 3.2.1 Point Feature

The 3D ear model consists of a number of points in 3D coordinates. Therefore, if the key points that are stable for the same ear and distinguishable for different ears could be found, then the 3D ear models would be recognized using these key points.

The aim of key-point detection is to select points on the 3D ear surface that can be identified with high repeatability in different models of the same surface. Islam and Mian proposed a key-point detection and feature extraction method that is effective on 3D ears [21] and faces [29]. Although the core of our key-point detection technique is similar to theirs, the technique is modified to make it suitable for the 3D ears data captured by our proposed device. In addition, the point feature is defined differently.

The input to the algorithm is a point cloud of the ear *E* = {*P*_1_,…,*P*_*n*_}. For each point *P*(*x*_*i*_ *y*_*i*_ *z*_*i*_)^*T*^, where *i* = 1,…,*n*, a local surface is cropped from the point cloud using a sphere of radius *r* centered at *P* and recorded as $SetL=\{{\left[x_{j}\;y_{j}\;z_{j}\right]}^{T}|\sqrt{{\left(x_{j}-x_{i}\right)}^{2}+{\left(y_{j}-y_{i}\right)}^{2}+{\left(z_{j}-z_{i}\right)}^{2}}<r\}$. The principle component analysis is then applied on the data points *SetL*. The difference between the eigenvalues along the first two principal axes of the local surface is computed as *d*. The value of *d* indicates the extent of asymmetry around the center point *P*, which is zero if *SetL* is planar or spherical. It is then compared to a threshold *t*, and if *d* > *t*, the point *P*(*x*_*i*_ *y*_*i*_ *z*_*i*_)^*T*^ is selected as a key-point. At the same time, the angular separation *φ* between the third principal axes and the original unified *Z* coordinate was calculated. Let *K*_*m*_ = [*x*_*m*_ *y*_*m*_ *z*_*m*_ *d*_*m*_ *φ*_*m*_]^*T*^ (where *m* = 1,…,*n*_*k*_) record the key-point information. Set *K*_*m*_ is used at a later stage of feature extraction. Parameters *r* and *t* are empirically chosen as *r* = 5 mm and *t* = 2 mm.

Fig 8 shows examples of key-points detected on four different point clouds scanned from two individuals. It illustrates that key-points are stable in the ear data of the same individual, and distinguishable for the ear data of different individuals.

**Fig 9:**
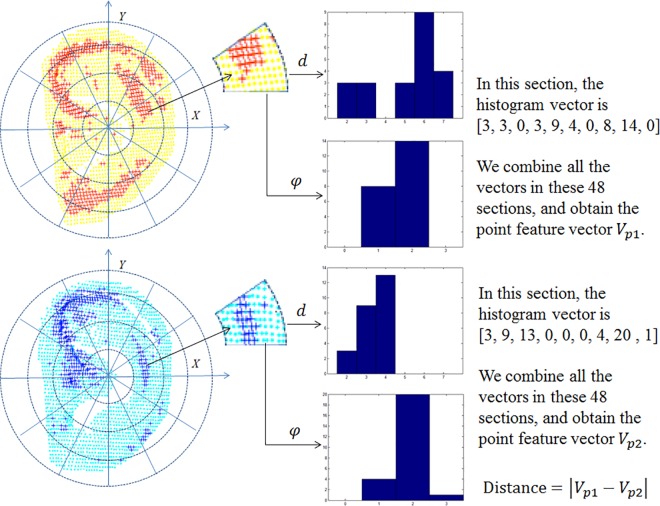
Extraction of the point feature vector.

After key-point detection, features are extracted from set *K*_*m*_ (as shown in Fig 9).

First, the normalized ear was divided into 12 average fan-shaped parts, where each sector is further divided into four equidistant parts. Each part is marked as *F*_*l*_, where *l* = 1,…,48. Thus, the *x-y-z* values of key-point set *K*_*m*_ fall within these 48 parts.

Second, for each *F*_*l*_, the statistical histograms of *d* and *φ* were calculated. The histogram bins of *d* are set to 2, 3, 4, 5, 6, and 7, and the bins of *φ* are set to 0, 1, 2, and 3. Next, the number in each bin was counted to obtain a 10-dimensional vector. If there is no key-point in *F*_*l*_, the vector was set to [0,0,0,0,0,0,0,0,0,0].

Finally, all 48 vectors were connected to obtain a 480-dimensional vector *V*_*p*_ as the final point feature vector. The difference between two ears is calculated using the Euclidean distance between their *V*_*p*_ vectors.

**Fig 10:**
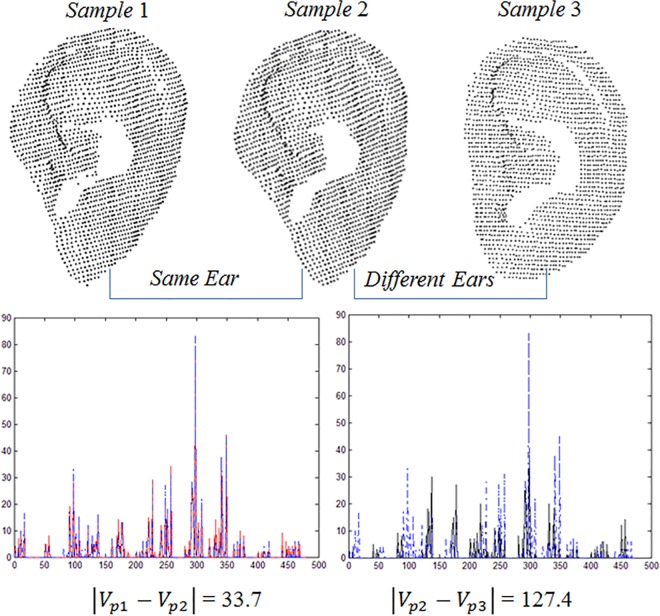
Discriminating between the same and different ears using the point feature vector.

Fig 10 shows the point feature vectors extracted from different samples. Sample 1 (S1) and 2 (S2) are from the same ear, and Sample 3 (S3) is from a different ear. The red curve is the point feature vector of S1, the blue curve is the point feature vector of S2, and the black curve is the point feature vector of S3. The distance between S1 and S2 is 33.7, and the distance between S1 and S3 is 127.4. It can be seen that the point feature vectors from the same ear are very similar, and those from different ears are dissimilar.

#### 3.2.2 Line Feature

**Fig 11:**
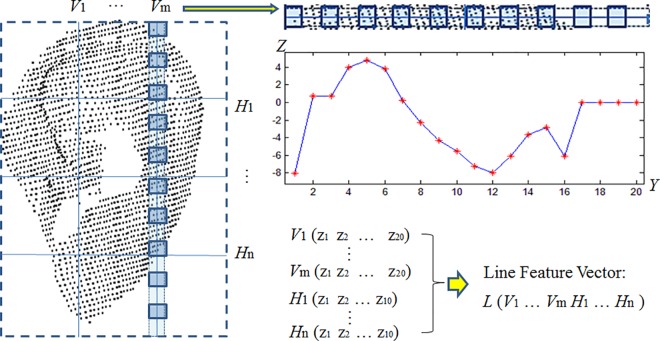
Extraction of the line feature vector.

To calculate the line feature, a rectangle was fitted on the normalized ear in the *X-Y* coordinates, define (*M* + *N*) lines, *V*_1_, …, *V*_*m*_ (which divides the rectangle equally in the horizontal direction), and *H*_1_, …, *H*_*n*_ (which divides the rectangle equally in the vertical direction), as shown in Fig 11. Next, the 3D points on each line were obtained and their *z* values were recorded. Each line was then divided equally and the *z* crossing point values were marked as *z*_*1*_, *z*_*2*_, …, *z*_*10*_ (or *z*_*1*_, *z*_*2*_, …, *z*_*20*_ for *V*_1_, …, *V*_*m*_). These *z* values were used to form the line feature vector *L* (*V*_1_, …, *V*_*m*_, *H*_1_, …, *H*_*n*_), where the vector is of length (20 × m + 10 × n).

**Fig 12:**
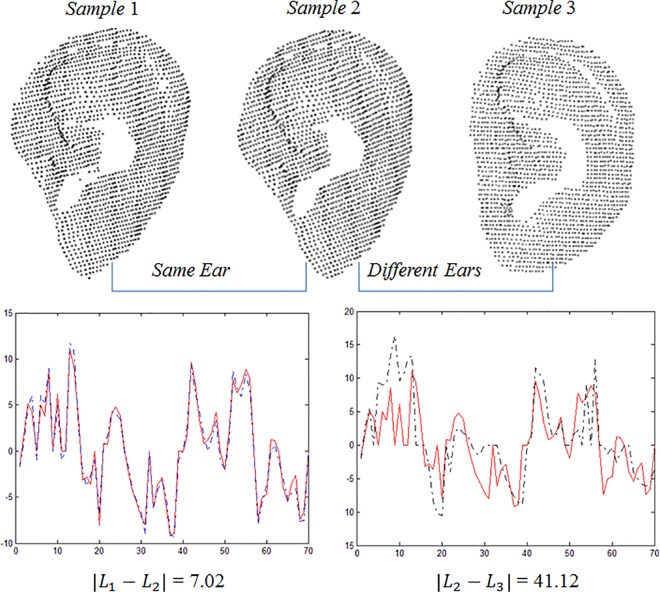
Discriminating between the same and different ears using the line feature vector.

Fig 12 shows the line feature vectors extracted from the same samples as those in Fig 11. Parameters *m* = 2 and *n* = 3 were used in the experiment to test the discrimination of the line feature. The distance between S1 and S2 using the line feature is 7.02, and the distance between S1 and S3 is 41.12. It can be seen that the line feature vectors from the same ear are very close, and the line feature vectors from different ears are further apart.

#### 3.2.3 Area Feature

**Fig 13:**
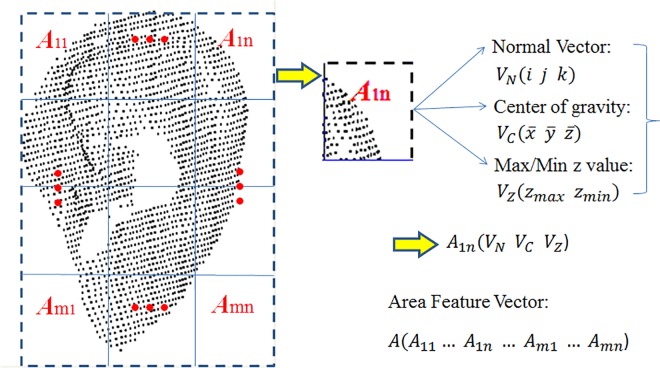
Extraction of the area feature vector.

In order to compute the area feature, the 3D ear was fitted into a fixed block and divided into m × n equal areas (see Fig 13). All coordinate points in the area are defined as (*x*_*i*_,*y*_*i*_,*z*_*i*_) *i* = 1,…,*N*, where *N* is the number of the points in the area. All the coordinates of these points constitute an N × 3 matrix *W* as follows:

$$W=\left[\begin{array}{ccc} x_{1} & y_{1} & z_{1}\\ & \ldots & \\ x_{N} & y_{N} & z_{N} \end{array}\right]$$ (4)

Principle component analysis [30, 31] is performed on *W* and the resulting normal vector is represented as *V*_*N*_(*i*,*j*,*k*).

The average is calculated using

$$(\bar{x},\bar{y},\bar{z})=\frac{1}{N}\sum_{i=1}^{N}\left(x_{i},y_{i},z_{i}\right)$$ (5)

**Fig 14:**
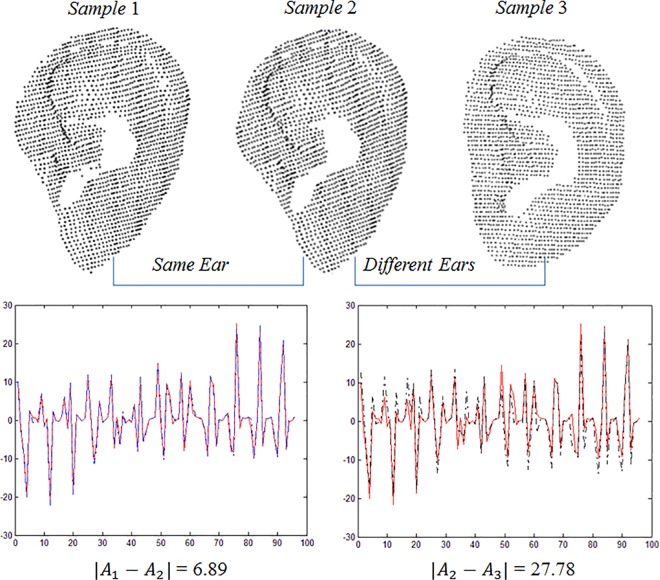
Discriminating between the same and different ears using the area feature vector.

The scatter matrix is given as $S=\sum_{i=1}^{N}(W_{i}-{\bar{W}}_{i})\times {\left(W_{i}-{\bar{W}}_{i}\right)}^{T}$, the eigenvectors of *S* are Φ, and the first column of Φ is the normal vector *V*_*N*_(*i*,*j*,*k*). It is clear that *V*_*N*_(*i*,*j*,*k*) can be thought of as the direction of matrix *W*. In addition, the center of gravity of *W* can be represented as $V_{C}(\bar{x},\bar{y},\bar{z})$. As a result, the normal vector V_N_, center of gravity V_C_, and min/max z values V_Z_ are calculated and joined to form a vector A_N_ for each area. The area feature subsequently becomes the vector consisting of all m × n vectors A, (A_11_, A_12_, …, A_mn_). Fig 14 shows the area feature vectors extracted from S1, S2, and S3. The distance between S1 and S2 is 6.89, and the distance between S2 and S3 is 27.78, which indicates that the area feature vectors from the same ear are alike and those from different ears are not alike.

## 4. Experimental Results and Discussion

The experiments were divided into two parts: feature optimization and verification experiments. As mentioned above, our database contains a total of 2,000 different samples from 500 individual ears. A PC with Intel Core 2 CPU @2.33 GHz and 2 GB memory was used in our experiments.

### 4.1 Feature Optimization

Because the parameters used in the definition of each local feature may influence the length of the feature vector as well as the equal error rate (EER) of the verification experiments, the feature optimization experiments were performed to determine the most effective values for these parameters.

**Fig 15:**
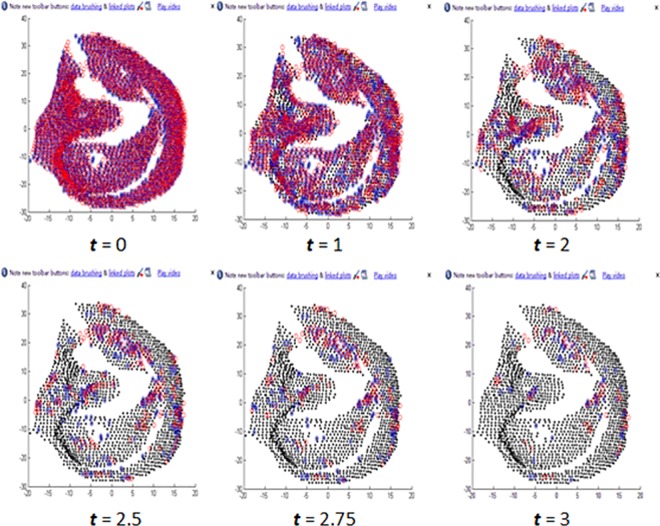
Point feature optimization.

In our point feature, the number and distribution of the key-points determines the point feature vector. Hence, threshold *t* is the parameter that needs to be optimized. Fig 15 shows the different key-points extracted using different thresholds, while Table 2 shows the EER for different thresholds. Considering the time consumed, the feature optimization experiments were performed on a sub-dataset that contains 100 different sample ears. From Table 2, it can be seen that the best result is achieved when *t* = 2.

**Table 2:** Point features with different *t* parameters.

***t***	0	1	2	2.5	2.75	3
**No. Points**	2009	1320	462	207	136	82
**EER (%)**	2.5	2.2	2.2	5.3	7.8	17.2

**Fig 16:**
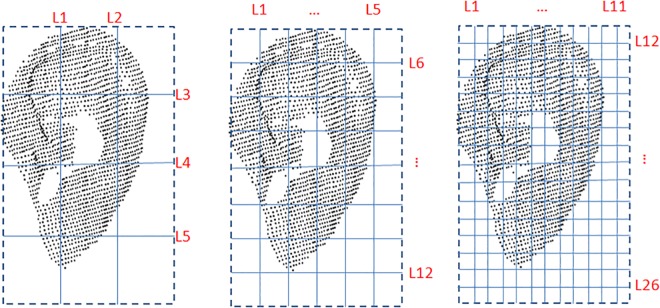
Line feature optimization.

The line feature vector is determined by the number of horizontal and vertical lines. Therefore, the line number is the parameter that needs to be optimized here. Fig 16 shows the different lines across the ear. Table 3 shows the EER obtained using different line numbers, where 12 lines obtains the lowest EER.

**Table 3:** Line features for different line numbers.

***Line No*.**	5	12	26
**Vector Length**	70	170	370
**EER (%)**	3.6	3.0	3.2

**Fig 17:**
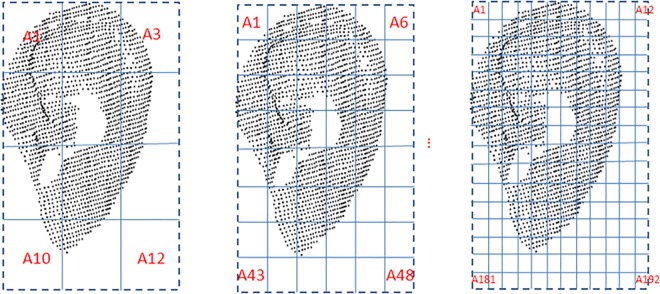
Area feature optimization.

Because the number of blocks determines the area feature vector, this parameter is the one that must be optimized. Fig 17 shows the different blocks on the ear and Table 4 shows the EER obtained using different block numbers. It can be seen that the best result is achieved when there are 48 blocks.

**Table 4:** Area features with different block numbers.

***No*. *Blocks***	12	48	192
**Vector Length**	108	432	1728
**EER (%)**	5.0	4.3	4.3

### 4.2 Matching Using Local Features

The matching experiments were carried on all 2,000 samples, and performed using the local feature class (point, line, and area features) as well as their feature-level fusion. Since all the local features (point, line, and area features) are defined in form of vectors (*V*_*P*_, *V*_*L*_, *V*_*A*_), the most direct strategy for feature-level fusion is to joint different vectors into one fusion feature vector. Therefore, the fusion feature vectors can be described as follows:

$$\left\{ \begin{array}{l} \;V_{P+L}=normalization\left(joint\left(normalization\left(V_{P}\right),normalization\left(V_{L}\right)\right)\right)\\ \;V_{P+A}=normalization\left(joint\left(normalization\left(V_{P}\right),normalization\left(V_{A}\right)\right)\right)\\ \;V_{L+A}=normalization\left(joint\left(normalization\left(V_{L}\right),normalization\left(V_{A}\right)\right)\right)\\ V_{local}=normalization\left(joint\left(normalization\left(V_{P+L}\right),normalization\left(V_{A}\right)\right)\right) \end{array}\right.$$ (6)

The function *normalization* normalizes the feature vector into unit vector. The function *joint* combines two feature vectors into one fusion feature vector. Table 5 shows the EER results of different local features and their combinations. It can be seen that the optimal result is achieved when all local features are fused together.

**Table 5:** Matching results for different local features.

Features	Point	Line	Area	Point + Line	Point + Area	Line + Area	All Features
**EER (%)**	4.7	4.2	5.1	3.3	4.5	3.6	2.8

### 4.3 Recognition with Global Feature Indexing

**Fig 18:**
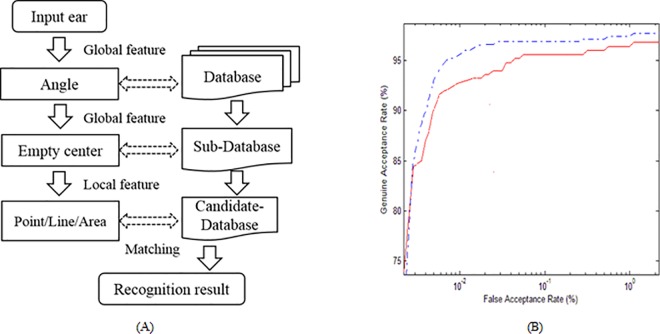
Fusion of global and local features: (A) flowchart of recognition with global feature indexing and (B) receiver operating characteristic curve of the global and local feature fusion.

Different from the weighted fusion method, the global and local features fusion is implemented in a hierarchical procedure. The 3D ears are pre-classified using global features and then recognized using local features. Thus, much time can be saved and accuracy can be improved in 3D ear recognition. The flowchart of the overall recognition with global feature indexing is shown in Fig 18A. For a given ear sample, the procedure is as follows:

1. Extract the global features of the test sample Angle(Gt), Center(Gt).
2. Compare Angle(Gt) with global features Angle(Gi) i = 1, …, N of all ear models (in our experiments, N = 500) to obtain the matching distance Dist(Angle(Gt), Angle(Gi)).
3. If Dist(Angle(Gt), Angle(Gi)) is smaller than threshold T(β), the ear model is treated as a matched candidate and place it into a sub-database.
4. Match test ear Gt with the sub-database ears using the empty center feature and adjust the candidate sub-database Gi accordingly.
5. Extract the local features of VLocal_t and the local features of the ear models in the candidate sub-database VLocal_i (i = 1,…,k), where k is the total number of ears it contains.
6. Match local features between VLocal_t and VLocal_i to measure the differences between the test ear and candidate ears (in our experiments, the Euclidean distance was used).
7. The candidate ear that is closest to the test ear is the recognition result.

Fig 18B shows the receiver operating characteristic curve of the results obtained by combining both global and local features together, where the EER is 2.2%. It can be seen that the fusion of global and local features achieves the smallest EER of all schemes, and is even better than single feature matching. This is reasonable, because more information usually leads to more accurate recognition.

### 4.4 Performance Analysis

To better measure the performance of the proposed method, six criteria (database, acquisition device, feature extraction method, average matching time, EER, and online properties) were used to compare the proposed method with other 3D ear recognition methods.

The results are shown in Table 6.

**Table 6:** Comparison with existing 3D ear recognition methods.

Reference	Dataset Size (ID/subjects/images)	Device Used	Method Applied	Matching Time (s)	Reported EER	Online
Yan and Bowyer	UND/415/1386	Vivid 910	ICP	1.5	1.2%	N/A
Chen and Bhanu	UND-F/302/942 UCR/155/902	Vivid910 Vivid300	LSP	3.7	2.3% 4.2%	N/A
Islam et al.	UND-F/302/942 UND-J/415/830	Vivid 910	L3DF + ICP	0.06	2.3% 4.1%	N/A
Proposed Method	HIT/500/2000	Our Scanner	Global + Local	0.5	2.2%	YES

From Table 1 and Table 6, it can be seen that our 3D ear scanner has a lower price (approximately 5% that of the Vivid 910), and a smaller size (approximately 25% that of the vivid 910). Meanwhile, the overall recognition time (including acquisition and recognition time) is less than 2.5 s, and the EER on a database with 2,000 samples is 2.2%. So far, our 3D ear recognition system is the only system offering an overall solution for both 3D ear data acquisition and optimized recognition. Its performance is sufficient to meet the online system requirements for a real-time application.

## 5. Conclusions

In this paper, two novel feature classes, global and local features, were defined and extracted from 3D ear point clouds. The global feature class includes the empty center and ear-parotic area angle, whereas the local feature class consists of point, line, and area features. The experimental results show that all features are stable for the same ear and distinguishable between different ears. Furthermore, global features can be used for indexing, while the combination of both global and local features produces matching results with an EER of 2.2% on our 3D ear database of 2,000 samples. Using our own developed scanner and the optimized recognition method, a real-time 3D ear recognition system is achieved.
